# SET SUMOylation promotes its cytoplasmic retention and induces tau pathology and cognitive impairments

**DOI:** 10.1186/s40478-019-0663-0

**Published:** 2019-02-15

**Authors:** Min Qin, Honglian Li, Jian Bao, Yiyuan Xia, Dan Ke, Qun Wang, Rong Liu, Jian-Zhi Wang, Bin Zhang, Xiji Shu, Xiaochuan Wang

**Affiliations:** 1 Department of Pathophysiology, School of Basic Medicine, Key Laboratory of Education Ministry of China for Neurological Disorders, Tongji Medical College, Huazhong University of Science and Technology, Wuhan, 430030 China; 2 Department of Histology and Embryology, School of Basic Medicine, Tongji Medical College, Huazhong University of Science and Technology, Wuhan, 430030 China; 3 Department of Pathology and Pathophysiology, School of Medicine, Jianghan University, Wuhan, 430056 China; 4 Co-innovation Center of Neuroregeneration, Nantong University, Nantong, 226001 JS China; 5 Department of Genetics and Genomic Sciences, Icahn Institute of Genomics and Multiscale Biology, Icahn School of Medicine at Mount Sinai, 1470 Madison Avenue, New York, NY 10029 USA

**Keywords:** Alzheimer’s disease, SET SUMOylation, PP2A, Tau hyperphosphorylation, Cognitive impairments

## Abstract

PP2A is a major regulator of tau phosphorylation, which is principally regulated by an endogenous nuclear protein inhibitor 2 of PP2A (I_2_^PP2A^), also named SET. However, how SET is post-translationally regulated and translocates from the nucleus to the cytoplasm remain incompletely understood. Here we show SET is SUMOylated at K68 residue that induces its cytoplasmic retention, resulting in Alzheimer disease (AD) like tau pathology and cognitive defects. SET is predominantly SUMOylated at K68 that leads to its translocation from the nucleus to the cytoplasm and subsequently induces inhibition of PP2A and hyperphosphorylation of tau in HEK-293 cells. Moreover, overexpression of wild type SET significantly inhibits PP2A activity, leading to tau hyperphosphorylation, less synapse loss and cognitive deficits. Conversely, blocking SET SUMOylation via mutating Lys 68 to Arg rescues tau pathology and cognitive impairments in C57/BL6 mice infected with adeno-associated virus encoding SET. Further, β-amyloid exposure of rat primary hippocampal neurons induces a dose-dependent SUMOylation of SET. Our findings suggest that SET SUMOylation stimulates its cytoplasmic retention and inhibits PP2A activity, consequently leading to tau hyperphosphorylation and cognitive impairments, which provides a new insight into the AD-like tau pathology.

## Introduction

Alzheimer’s disease (AD) is the most common neurodegenerative disorder [5], which is characterized by the presence of two major neuropathological alterations: extracellular senile plaques consisting of β-amyloid (Aβ) and intracellular neurofibrillary tangles (NFTs) made up of the abnormally hyperphosphorylated tau [12, 15]. Although the triggering mechanisms of AD pathogenesis are still unclear, it is clinically recognized that the severity of dementia is positively correlated with tangle load and the spatial brain distribution in AD patients [2, 28]. The inhibition of protein phosphatase 2A (PP2A) activity leads to tau hyperphosphorylation, considered as the main driver for the formation of NFTs [22], which is widely expressed in different tissues and localizes primarily in the nucleus [33], where it mainly protects histones from acetylation [29]. Previous studies have demonstrated that SET translocates from the nucleus to the cytoplasm in AD patients’ brain where it is retained to down-regulate PP2A activity [26, 34, 36]. However, the specific mechanisms that lead to SET cytoplasmic retention are thus far unclear.

SUMOylation is an important posttranslational modification. In humans, SUMO-1, SUMO-2, and SUMO-3 are small ubiquitin-like proteins and the main members of SUMO family. SUMO conjugation and binding to target proteins regulates a wide variety of important cellular pathways [24, 25, 31]. The functional aspects of SUMOylation include changes in protein-protein interactions, intracellular trafficking, protein aggregation and degradation [10, 17, 18, 27, 35, 38]. An increasing number of studies suggest the association of SUMOylation with AD progression and an increase of SUMO-1 level in AD brain [19]. Studies have shown that tau and amyloid precursor protein (APP) are modified by SUMO [8, 9, 32, 41], whilst we have demonstrated that both beta-secretase (BACE1) at lysine 501 and tau at lysine 340 are the targets for SUMO-1 [3].

In this study, through investigating other proteins related to AD that can be modified by SUMO, SUMOsp2.0 software predicted SET as a target, due to the presence of an ^67^LKVE^70^ consensus sequence with a high probability for SUMO-1. Herein, we confirm that both in vivo and in vitro SET can be modified by SUMO-1 and its modification site is K68 residue. Following SUMO-1 modification, SET is retained in the cytoplasm and inhibits the activity of PP2A, resulting in tau hyperphosphorylation. SET SUMOylation and subsequent PP2A inhibition therefore influence downstream events in AD, and contribute to loss of synapse, learning and memory dysfunction.

## Materials and methods

### Plasmids, viruses, chemicals and antibodies

pcDNA3.1(+)SET, pcDNA3.1(+)SET-K14R, pcDNA3.1(+)SET-K36R, pcDNA3.1(+)SET-K39R, pcDNA3.1(+)SET-K68R, and pIRES2-UBE21(UBC9)-Myc, were constructed using Obio Technology(Shanghai)Corp., Ltd. AAV2-pCAG-SET WT, AAV2-pCAG-SET K68R were constructed and packaged using Obio Technology (Shanghai) Corp., Ltd. Nuclear and Cytoplasmic Extraction Reagents were purchased from Sigma Aldrich. Lipofectamine 2000 transfection reagent was purchased from Thermo Fisher Scientific. CELLBANKER 2 serum-free cell cryopreservation solution was purchased from Nippon Zenyaku Kogyo Co., Ltd. Geneticin (G418) was purchased from Thermo Fisher Scientific.

All antibodies used in the study are listed in Table 1.

**Table 1:** Antibodies employed in this study

Antibody	Specificity	Type	Dilution	Source
Flag	Detecting Flag fusion sequence	mAb	1:1000 for WB 1:200 for IP	Sigma
SET	Detecting I_2_^PP2A^ of mouse, rat and human origin	mAb	1:1000 for WB 1:50 for IP	Santa Cruze
SUMO-1	Detecting SUMO-1 and endogenous levels of SUMOylated proteins	mAb	1:1000 for WB 1:50 for IP	Cell Signaling
UBC9	Detecting endogenous levels of total UBC9 protein	mAb	1:1000 for WB	Cell Signaling
PP2A	Serine/threonine-protein phosphatase 2A catalytic subunit alpha isoform	mAb	1:1000 for WB	Millipore
Tau-5	Total-tau	mAb	1:1000 for WB	Lab Vision
pS199	Phosphorylated Tau at Ser199	pAb	1:1000 for WB	Invitrogen
pS396	Phosphorylated Tau at Ser396	pAb	1:1000 for WB	Invitrogen
pS404	Phosphorylated Tau at Ser404	pAb	1:1000 for WB	SAB
Synapsin-1	Detecting Synapsin-1 protein	mAb	1:1000 for WB	Millipore
Synaptophysin	Recognizing synaptophysin in mice brain extracts	mAb	1:1000 for WB	Sigma
Synaptotagmin	Recognizing synaptotagmin in mice brain extract	mAb	1:1000 for WB	Abcam
PSD95	Detecting endogenous levels of total PSD95 protein	mAb	1:1000 for WB	Cell Signaling
PSD93	Detecting endogenous levels of total PSD93 protein	mAb	1:1000 for WB	Cell Signaling
NR2A	N-methyl D-asparate receptor subtype 2A	mAb	1:1000 for WB	Millipore
GluR1	Detecting the Glutamate receptor 1 protein	mAb	1:1000 for WB	Millipore
DM1A	Recognizing alpha-tubulin	mAb	1:1000 for WB	Sigma

*mAb* Monoclonal antibody, *pAb* Polyclonal antibody, *WB* Western blot, *IP* Immunoprecipitation, *IF* Immunofluorescence, *PP2A* Protein phosphatase-2A

### Cell culture and transfection

Human embryonic kidney 293 (HEK293T) cells were cultured in Dulbecco’s modified eagle’s medium (DMEM) supplemented with 10% fetal bovine serum (FBS, Gibco BRL, Gaithersburg, MD, USA). HEK293/tau cells which were stably transfected with the longest human tau (tau441) were cultured in Dulbecco’s modified Eagle’s medium (Gibco, Invitrogen; Bleiswijk, Netherlands) in the presence of 200 mg/mL G418 containing 10% fetal bovine serum, and in a humidified incubator aerated with 95% air and 5% CO_2_ at 37 °C. Cells were seeded in 6-well or 12-well culture plates for 24 h and co-transfected using 1.8 μg of plasmid and 4 μl Lipofectamine 2000 (Invitrogen, Carlsbad, CA, USA) according to the manufacturer’s protocols. Cells were collected for subsequent experiments 48 h post-transfection. UBC9 is the only SUMO conjugating enzyme and plays an important role in targeting SUMOylation consensus site [37]. UBC9 can be detected both in vivo and in vitro, but the amount of expression is very low [7]. In order to increase the level of SUMOylation, we overexpressed UBC9 to increase the level of SUMOylation of SET.

### Primary cultures of hippocampal neurons

Primary cultures of rat hippocampal neurons were prepared from E18 Sprague–Dawley rat embryos as previously reported [23]. Briefly, hippocampi were dissected in D-Hanks supplemented with glucose (18 mM). Then mechanically dissociated in minimum essential medium (MEM) and seeded onto poly-L-lysine (25 μg/mL) coated six-well plates at a density of 8 × 10^4^ cells per well in neurobasal medium containing 2% (vol/vol) B-27, glutamax (2 mM), penicillin (50 U/mL), and streptomycin (50 μg/mL) (Gibco). After 4–6 h, planting medium was replaced with neurobasal medium containing 2% (vol/vol) B-27, GlutaMAX (2 mM), penicillin (50 U/mL), and streptomycin (50 μg/mL) (Gibco). Media was half-changed every 3 days. Hippocampal neurons were cultured for 14 d at 37 °C in a humidified 5% (vol/vol) CO_2_ incubator before treatment .

### Aβ treatment

Human Aβ1–42 peptide were purchased from Abcam (beta-Amyloid Peptide (1–42) (human) (ab120301)). Peptides were dissolved in sterile water at a concentration of 100 μM and stored at 4 °C. Peptide solutions were incubated at 37 °C for 1 h before experimental use and were found to occur in a predominantly oligomeric form [14]. To investigate the effect of Aβ on SET SUMOylation, Aβ was added to the cell culture at 14 DIV for 24 h. Then the samples were subjected to immunoprecipitation and western blotting.

### Animals

3 × Tg AD mice (PS1m146v/APPswe/TauP301L) were purchased from the Jackson Laboratory. APP/PS1 mice were from the Model Animal Research Center of Nanjing University. Male C57/BL6 mice (3-month old, 25 ± 2 g) were supplied by the Experimental Animal Central of Wuhan University. All the animals were housed in an air conditioned room (22 ± 2 °C, 12-h light/dark cycle) with free access to food and water. Behavioral tests were performed during active hours.

### Immunoprecipitation analysis

Cells or mouse brain tissue samples were lysed with RIPA (50 mM Tris pH 7.4, 150 mM NaCl, 1% NP-40, 0.25% sodium deoxycholate, sodium orthovanadate, sodium fluoride, EDTA, leupeptin) and centrifuged for 15 min at 12,000 g. Supernatants were incubated with antibodies overnight and added to protein A/G-agarose at 4 °C. After 24 h, bound proteins were eluted from the beads by centrifugation for 15 min at 12,000 g and subjected to western blot analyses.

### Western blotting

Cell or mouse brain tissue samples were lysed with RIPA supplemented with protease inhibitor PMSF and cocktail (catalog number:P8340, from Sigma-Aldrich), and then centrifuged for 15 min at 12,000 g. Supernatants were boiled in SDS loading buffer and protein separated using SDS–PAGE. Proteins were then transferred to nitrocellulose membranes. Western blotting analysis was performed using the indicated antibodies.

### Assessment of PP2A activity

PP2A activity in cell and brain tissue samples was measured using the phosphatase kit V2460 according to the manufacturer’s procedure (Promega).

### Immunofluorescence

Cells or mouse brain slices were fixed in 4% paraformaldehyde for 20 min, washed with PBS, and blocked in 3% BSA and 0.5% Triton X-100 for 30 min. Cells were labeled via overnight incubation with primary-antibodies at 4 °C. Cells/brain slices were washed three times in PBS and incubated with the secondary antibodies conjugated to Alexa-Fluor 488/548 for 1 h at room temperature. Cells/brain slices were then washed in PBS and the nuclei were stained with Hoechst (1:1000) for 5 mins. Cells/slices were washed three times in PBS, and mounted by covering with glass coverslips using mounting solution. Cells were examined using confocal microscopy (LSM710, Zeiss, Germany).

### Open field testing

Each mouse was placed in a 38 × 42 cm open field from the fixed position, and the paths of mice movement were recorded by USB webcam and video capture software for 10 min. Total distances and time in the central area (15 cm × 15 cm, imaginary square) were recorded. After each mouse was tested, the open filed arena was cleaned with 70% ethanol and wiped with a paper towel.

### Fear conditioning test

Fear conditioning tests included two periods. The first period involved training: mice were placed in the chamber and sound stimulation was administered for 10 s after 3 min. Short-term current stimulation (0.8 mA, 2 s) immediately followed. The current stimulation cycle was repeated three times. After either 4 h or 24 h, the detection period was performed: This period involved only sound stimulation, with no current administered. Freezing times were then recorded. After each mouse was tested, the chamber was cleaned with 70% ethanol and wiped dry with paper.

### Morris water maze tests

Mice were trained in a round, water-filled tub in an environment rich with extra maze cues. An invisible escape platform was located in a fixed spatial location 1 cm below the water surface no matter where the mice began from. During each trial, the mice were placed in four fixed positions (N, S, E, W) of the water maze. Each interval lasted 15 min. The maximum test time was 60s. If mice could not find the platform within 60s, the tester manually guided the mouse to the platform on which the mouse was allowed to stay for 15 s. After consecutive training for 5 days, the platform was removed and on the sixth day, the number of times the mice crossed the original platform in 60 s was recorded. The temperature of the water was monitored each day to ensure mice were tested in water between 22 °C and 25 °C. Once the experiments were completed, the mice were wiped dry.

### Statistical analysis

All data are shown as the mean ± SD and analyzed using a Student’s two-tailed, unpaired t-test. The level of significance was set at *P* < 0.05. All statistical analyses were performed using Prism 7 (GraphPad).

## Results

### SET is SUMOylated in vitro and in vivo

**Fig. 1:**
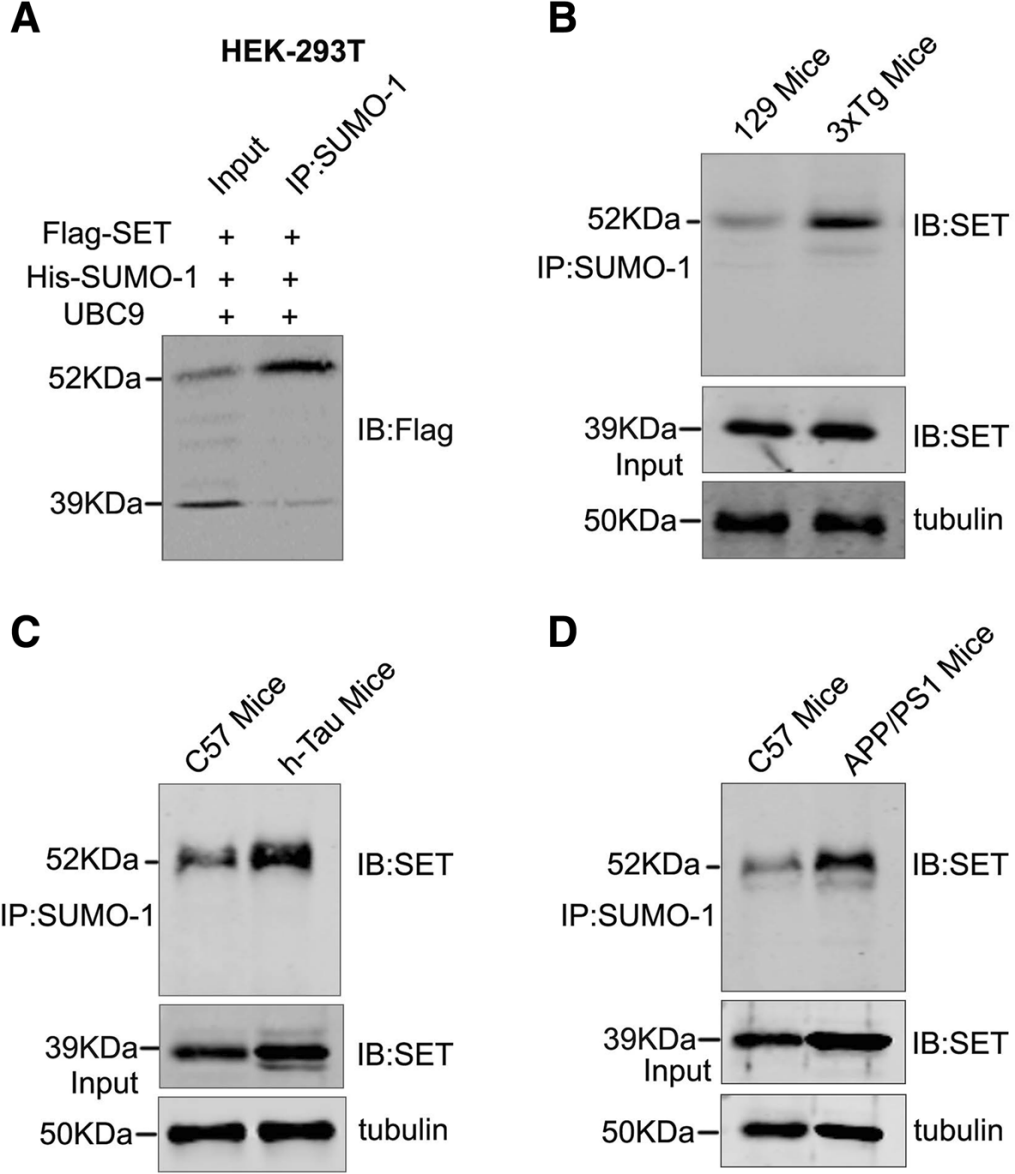
SET is SUMOylated in vitro and in vivo. **a** HEK-293 T cells were transfected with Flag-SET, His-SUMO-1 and UBC9 for 48 h. Cells were lysed and immunoprecipitations performed using anti-SUMO-1 antibodies. Pull-downs were subjected to western blotting analysis and probed with anti-Flag antibodies. **b**-**d** Hippocampal homogenates from 3 × Tg mice/h-tau mice (from the Jackson Laboratory, 3rd generation) /APP/PS1 mice (from the Model Animal Research Center of Nanjing University. 2nd generation) were harvested and immunoprecipitations performed using anti-SUMO-1 antibodies. Pull-downs were subjected to western blotting analysis and probed with anti-SET antibodies

To explore whether SET can be modified by SUMO-1, we co-transfected Flag-SET, His-SUMO-1, and UBC9 (SUMO-conjugating enzyme) plasmids into HEK-293 T cells. The results of co-immunoprecipitation experiments demonstrated that SET bound to SUMO-1 at 52 KDa (Fig. 1a). To further investigate that alteration of SET SUMOylation is associated with AD, we detected the level of SET SUMOylation of hippocampal homogenates in three kinds of AD mice and 129 or C57 mice as controls for confirmation of SET SUMOylation in a more physiologically relevant system. We found that SET bound to neuronal SUMO-1 in 129 and C57 mice (Fig. 1b-d), indicating its SUMOylation in neuronal cells. Moreover, we found that endogenous SET was also highly SUMOylated in 3 × Tg AD (Fig. 1b), h-Tau (Fig. 1c) and APP/PS1 (Fig. 1d) mice. Taken together, these data strongly demonstrate SUMO-1 modification of neuronal SET and hyper-SUMOylation of SET is related to AD.

### K68 residue is the major SET SUMOylation site

**Fig. 2:**
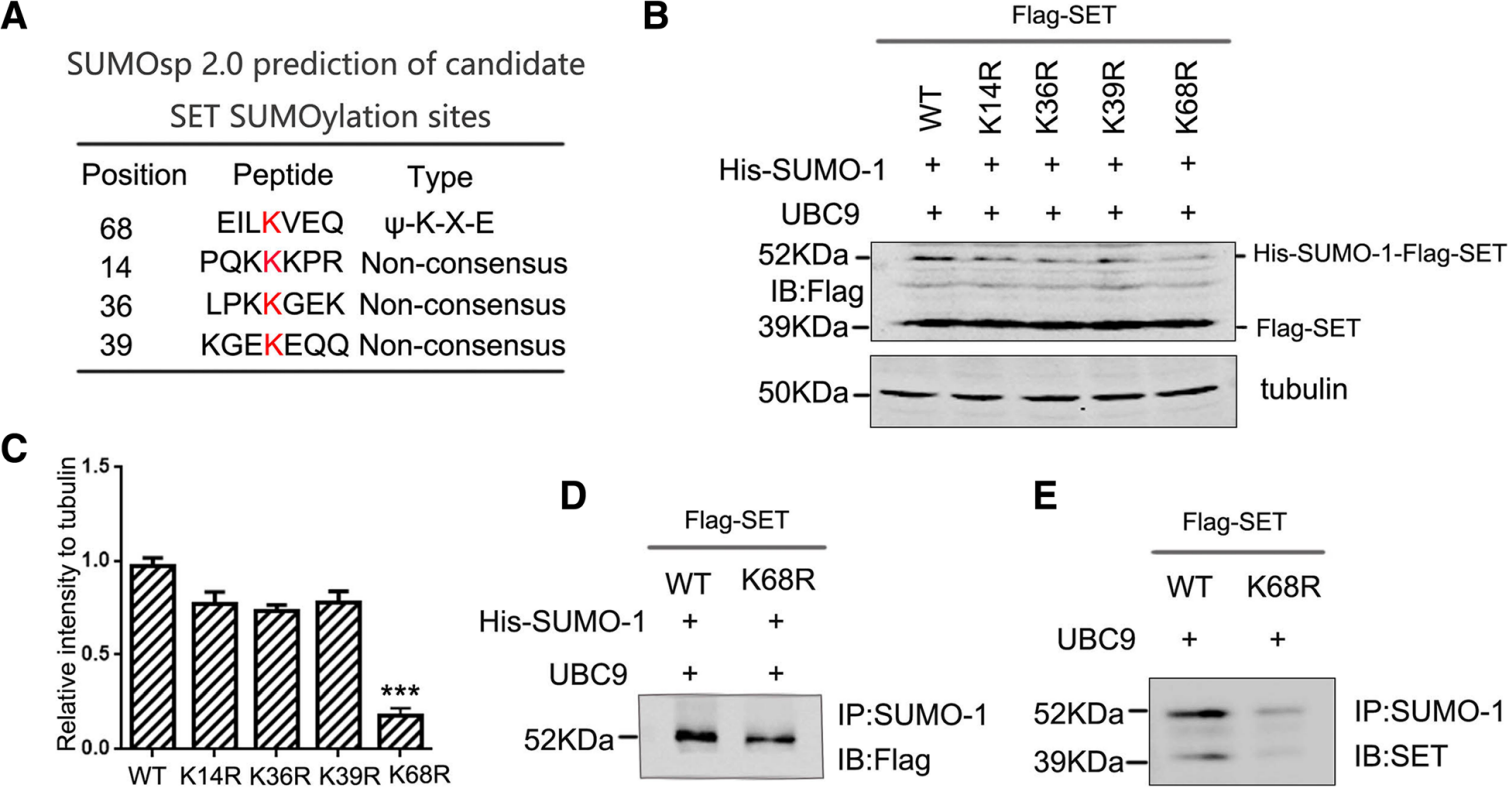
K68 is a major SET SUMOylation site. **a** SUMOsp2.0 prediction of candidate SET SUMOylation sites. **b** HEK-293 T cells were transfected with SET-WT, SET-K14R, SET-K36R, SET-K39R or SET-K68R. Cells were lysed and subjected to western blotting analysis with anti-Flag antibodies. **c** Quantification of the blots in B. ***P < 0.001 vs. WT. All data represent the mean ± SD of 3 independent experiments. **d** HEK-293 T cells were co-transfected with Flag-SET-WT, Flag-SET-K68R, His-SUMO-1 and UBC9 plasmids for 48 h. Cells were lysed and co-immunoprecipitations performed to detect SET SUMOylation. **e** HEK-293 T cells were co-transfected with Flag-SET-WT, Flag-SET-K68R and UBC9 for 48 h. Cells were lysed and immunoprecipitations performed using anti-SUMO-1 antibodies. Pull-downs were subjected to western blotting analysis and probed with anti-SET antibodies

In order to explore the sites of SET SUMOylation, we first used SUMOsp2.0 software to predict its potential SUMOylated sites. Four potential sites were identified: K14, K36, K39, and K68 (Fig. 2a). We mutated these four lysine residues into Arginine (R) to mimic non-SUMOylated SET and assessed SET-SUMO1 association. Flag-tagged SET/WT or mutants were transfected into HEK293T cells in the presence of UBC9, western blot developed with mAb Flag showed that K14R, K36R, and K39R showed a downward trend in SET-SUMO-1 interaction, but no significant difference compared to wild type SET. However, K68R mutant evidently impeded the SET-SUMO-1 interaction (Fig. 2b-c), suggesting that K68 might be the major SUMOylation site. To further validate this observation, we performed co-immunoprecipitation assay, and found that mutation of SET K68 to R induced an obvious decrease in SET binding to SUMO-1 as compared to SET-WT group (Fig. 2d). To confirm SET modification by endogenous SUMO-1, we transfected only Flag-SET-WT or Flag-SET-K68R and UBC9 into HEK-293 T cells. We also observed that SET interaction with endogenous SUMO-1 was significantly reduced with SET K68R (Fig. 2e). Therefore, these findings imply that SET can be modified by endogenous SUMO-1 at K68 residue.

### SUMOylation of SET at K68 induces its cytoplasmic retention

**Fig. 3:**
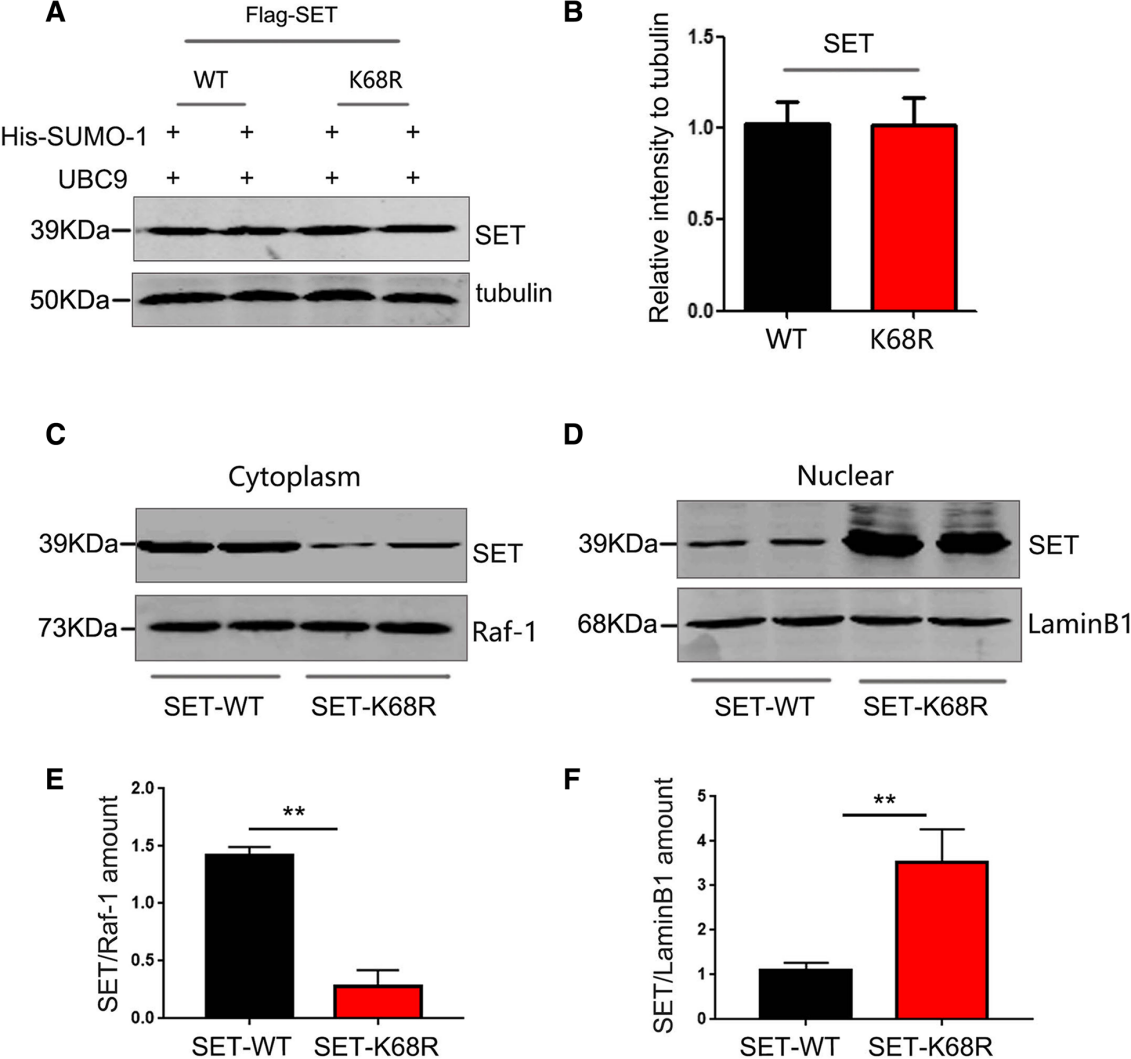
SUMOylation of SET at K68 induces its cytoplasmic retention. **a** HEK-293 T cells were co-transfected with Flag-SET-WT, Flag-SET-K68R, His-SUMO-1 and UBC9 plasmids for 48 h. Cells were lysed and probed for SET via western blotting analysis. **b** Quantification of the blots described in (**a**) was performed using ImageJ software. **c** and **d** Cytosolic and nuclear fractions were prepared in cells co-transfected with Flag-SET-WT or Flag-SET-K68R, His-SUMO-1 and UBC9 for 48 h. Levels of SET in the cytoplasm and nuclear were determined by western blotting with anti-SET antibodies. Relative purity of the fractions was confirmed by sequential probing for the cytoplasmic marker Raf-1 and the nuclear marker LaminB1. **e** and **f** Quantification of the blots in C-D. **P < 0.01

Our recent study has shown that phosphorylation of SET leads to its cytoplasmic translocation [40]. To investigate whether SET SUMOylation influences its intracellular distribution and translocation, we first assessed the effects of SUMOylation on the total protein levels of SET. In HEK-293 T cells co-transfected with Flag-SET-WT or Flag-SET-K68R with SUMO-1 and UBC9 plasmids. SET levels were assessed by western blotting analysis. The results revealed no differences in the total levels of SET among each group, suggesting that SUMOylation does not affect SET stability (Fig. 3a-b). We next examined the subcellular localization of SET via nuclear/cytoplasmic extractions 48-h post-transfection (Fig. 3c, d). We observed that SET-WT displayed higher levels of cytoplasmic expression whilst SET-K68R was more abundant in the nucleus (Fig. 3e, f). These results suggest that SET SUMOylation mediates its cytoplasm retention.

### SUMOylation of SET leads to PP2A inhibition and tau phosphorylation

**Fig. 4:**
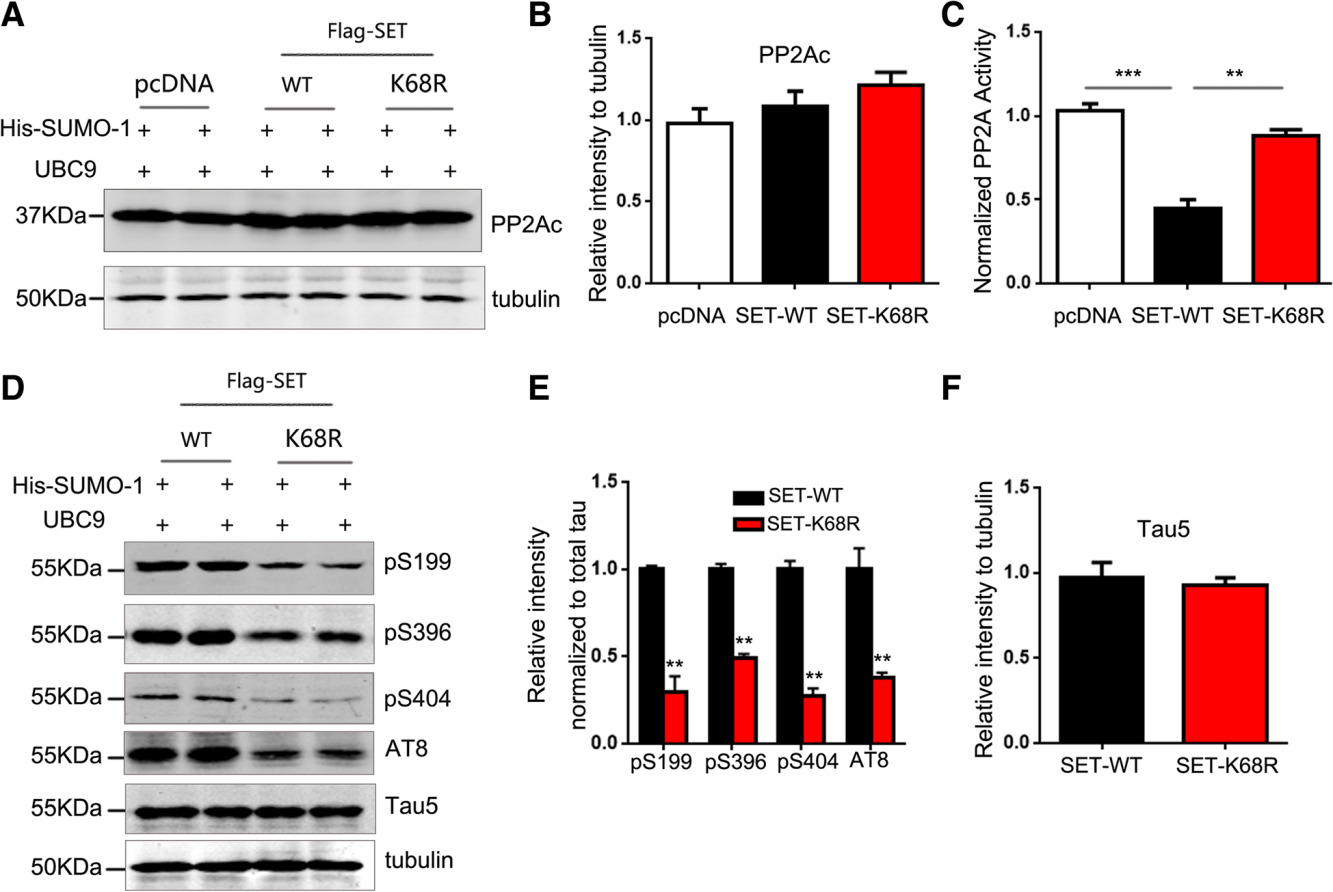
SUMOylation of SET inhibits PP2A and promotes tau phosphorylation. **a** HEK-293 T cells were transfected with pCDNA3.1 (+), Flag-SET-WT or Flag-SET-K68R, His-SUMO-1 and UBC9 plasmids for 48 h. Cells were lysed and probed for total levels of PP2A via western blotting. **b** Quantification of the blots described in (**a**) was performed using ImageJ software. **c** PP2A activity was measured using commercially available assays. **P < 0.01, ***P < 0.001. (*n* = 3). **d** Levels of tau phosphorylation at Ser396, Ser404, AT-8 (Ser202, Ser205) in transfected HEK-293tau cells were assessed by western blotting analysis and **e** quantified using ImageJ. ** *P* < 0.01 vs. SET-WT transfection control. **f** Total tau developed with Tau5 were comparable among each group. All data shown represent the mean ± SD of 3 independent experiments

Because SET is a specific inhibitor of PP2A [4], we next investigated the effects of SET SUMOylation on the activity of PP2A. In HEK-293 T cells co-transfected with Flag-SET-WT or Flag-SET-K68R with SUMO-1 and UBC9 plasmids, western blotting analysis showed comparable levels of PP2A expression (Fig. 4a, b), suggesting that SET SUMOylation does not influence PP2A stability. We then assessed PP2A activity in co-transfected cells. The activity of PP2A in cells co-transfected with Flag-SET-WT, SUMO-1, and UBC9 was significantly lower than untransfected controls, whilst the levels of PP2A activity were restored in the presence of Flag-SET-K68R, indicating that K68R mutant rescues PP2A activity (Fig. 4c). These results suggest that SET SUMOylation enhances PP2A inhibition.

PP2A is an important protein phosphatase that dephosphorylates tau. We assessed tau phosphorylation in HEK-293Tau cells exogenously expressing Flag-SET-WT or Flag-SET-K68R with SUMO-1 and UBC9 by western blotting analysis. We detected significantly higher levels of phosphorylated tau including Ser199, Ser 396, Ser404, AT-8 (Ser202/Ser205) in cells expressing SET WT compared to K68R mutant (Fig. 4d-e), while the level of total tau (Tau5) were comparable among each group (Fig. 4f). These results suggest that SET SUMOylation induces a decrease of PP2A activity, which in turn leads to hyperphosphorylation of tau.

### SUMOylation of SET induces cognitive deficits with tau pathology in mice

**Fig. 5:**
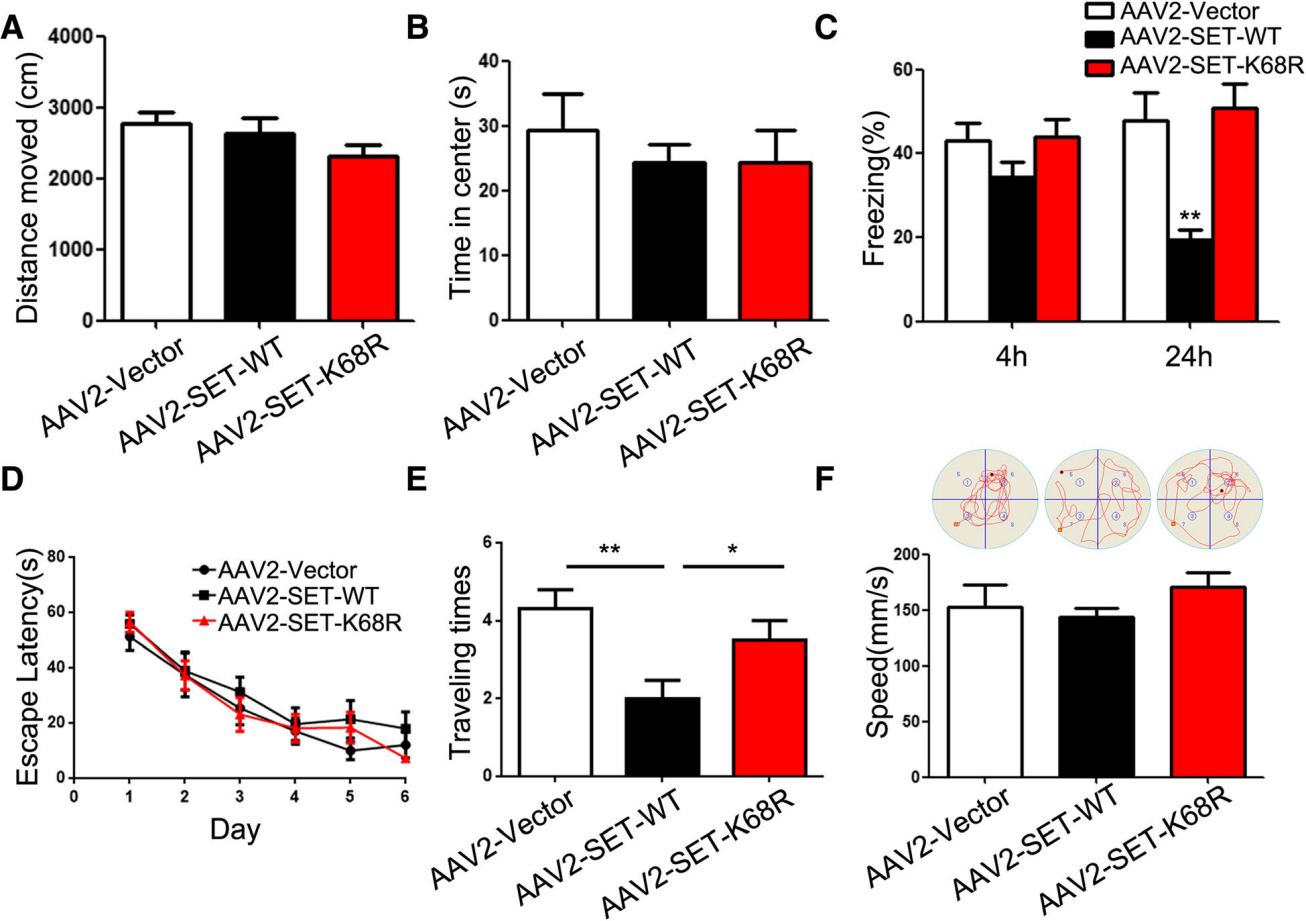
SUMOylation of SET leads to cognitive deficits in mice. **a** C57/BL6 mice were injected with pAAV-CAG-mCherry-2A-MCS-3 × FLAG-AAV2 (Vector-Titer = 1.0 × 10^12^), pAAV-CAG-mCherry-2A-SET-WT-3 × FLAG-AAV2 (SET-WT Titer = 1.0 × 10^12^) or pAAV-CAG-mCherry-2A-SET-K68R-3 × FLAG-AAV2 (SET-K68R Titer = 1.0 × 10^12^) as indicated in the bilateral hippocampus CA1 zone. Five weeks post-injection of viruses, **a** Total distances traveled and **b** Time in the center (15 × 15 cm imaginary square) were measured during open field test. **c** Fear conditioning was used to measure the contextual memory. Mice were allowed to adapt and the total percentage freezing in 3 min was measured at 4 h and 24 h. **P < 0.01 vs. AAV2-Vector. **d** Morris water maze test was employed to assess to learning and memory functions in the spatial reference memory task. No differences in escape latency among each group and **e** the number of times that each group of mice crossed the platform on day 7.*P < 0.05, **P < 0.01. **f** Mean swimming speed of each group on day 7

Learning and memory behavioral disorders are the clinical phenotypes of AD. To explore whether SUMOylation of SET influences learning and memory function, we conducted open field testing, contextual fear-conditioning testing and Morris water maze assessments in mice. Three -month-old C57/BL6 mice were injected with wild type SET (SET WT) or non-SUMOylated SET K68R (SET K68R) into the CA1 of hippocampus. One month later, open-field experiments were performed. Our results showed no significant differences in total distances moved or the time spent in the central area among each group (Fig. 5a, b), suggesting no significant differences in either autonomous behavior, exploratory behavior or tension among each group. The analysis of conditional fear testing showed no significant differences in percentage freezing times measured after 4 h, however the percentage freezing time in the AAV2-SET-WT group was significantly lower than that of the control group after 24 h. Non-SUMOylated SET K68R group displayed no significant changes compared to vector control (Fig. 5c). These results indicate that SET-SUMOylation can influence long-term memory function in mice without affecting short-term memory. We next used the Morris water maze to assess the spatial learning and memory abilities. After 6 days of training, no statistical difference in latency among the three groups of mice was observed (Fig. 5d). On the 7th day, we removed the platform and assessed the number of times that each group of mice crossed the platform. The statistical results showed that, compared to the control group, the number of mice crossing the platform in the AAV2-SET-WT group was significantly reduced (Fig. 5e). No such significant differences were evident for non-SUMOylated SET K68R group compared to the vector control (Fig. 5e). In addition, no significant differences in swimming speed between mice in each group was observed, indicating that SET or its non-SUMOylated mutant does not affect motor function (Fig. 5f). Taken together, these findings demonstrate that SET SUMOylation causes learning and memory dysfunction in mice.

**Fig. 6:**
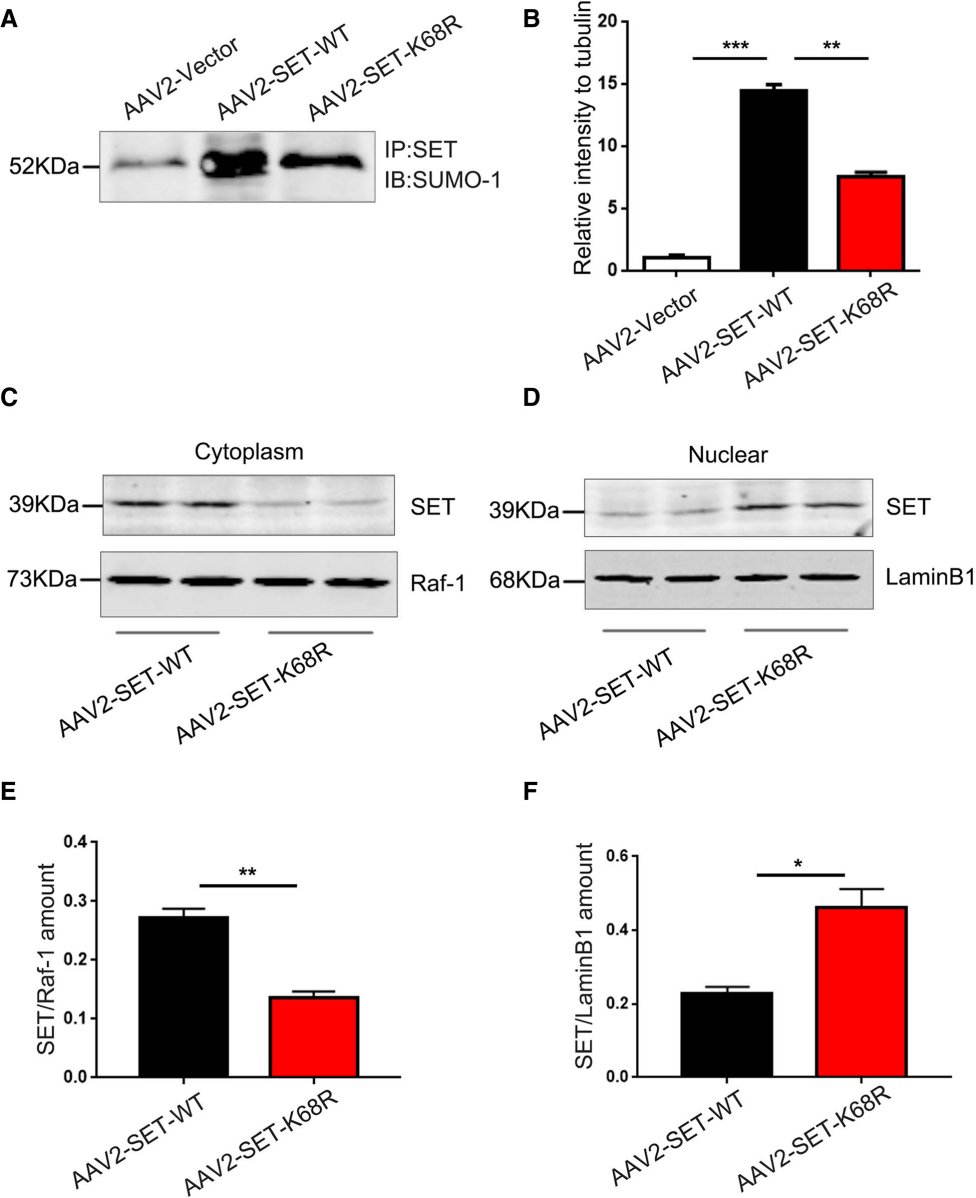
SET WT overexpression induces its SUMOylation and cytoplasmic retention. **a** C57/BL6 mice were injected with pAAV-CAG-mCherry-2A-MCS-3 × FLAG-AAV2 (Vector Titer = 1.0 × 10^12^), pAAV-CAG-mCherry-2A-SET-WT-3 × FLAG-AAV2 (SET-WT Titer = 1.0 × 10^12^) or pAAV-CAG-mCherry-2A-SET-K68R-3 × FLAG-AAV2 (SET-K68R Titer = 1.0 × 10^12^) as indicated in the bilateral hippocampus CA1 zone. Five weeks post-injection, hippocampal tissue was homogenized and immunoprecipitations performed using anti-SET antibodies. Pull-downs were immunoblotted with the SUMO-1 antibody. **b** Quantification of SET combined with SUMO-1 is shown. **P < 0.01, ***P < 0.001. **c** and **d** Levels of SET in nuclear and cytoplasmic fractions was determined by immunoblotting with anti-SET antibodies. The relative purity of fractions was confirmed by sequential probing for the nuclear marker laminB1 and the cytoplasmic marker Raf-1. **e** Quantification of SET in the cytoplasmic and **f** nuclear fractions are shown. *P < 0.05, **P < 0.01. Representative confocal images are shown. All data shown represent the mean ± SD of 3 independent experiments

**Fig. 7:**
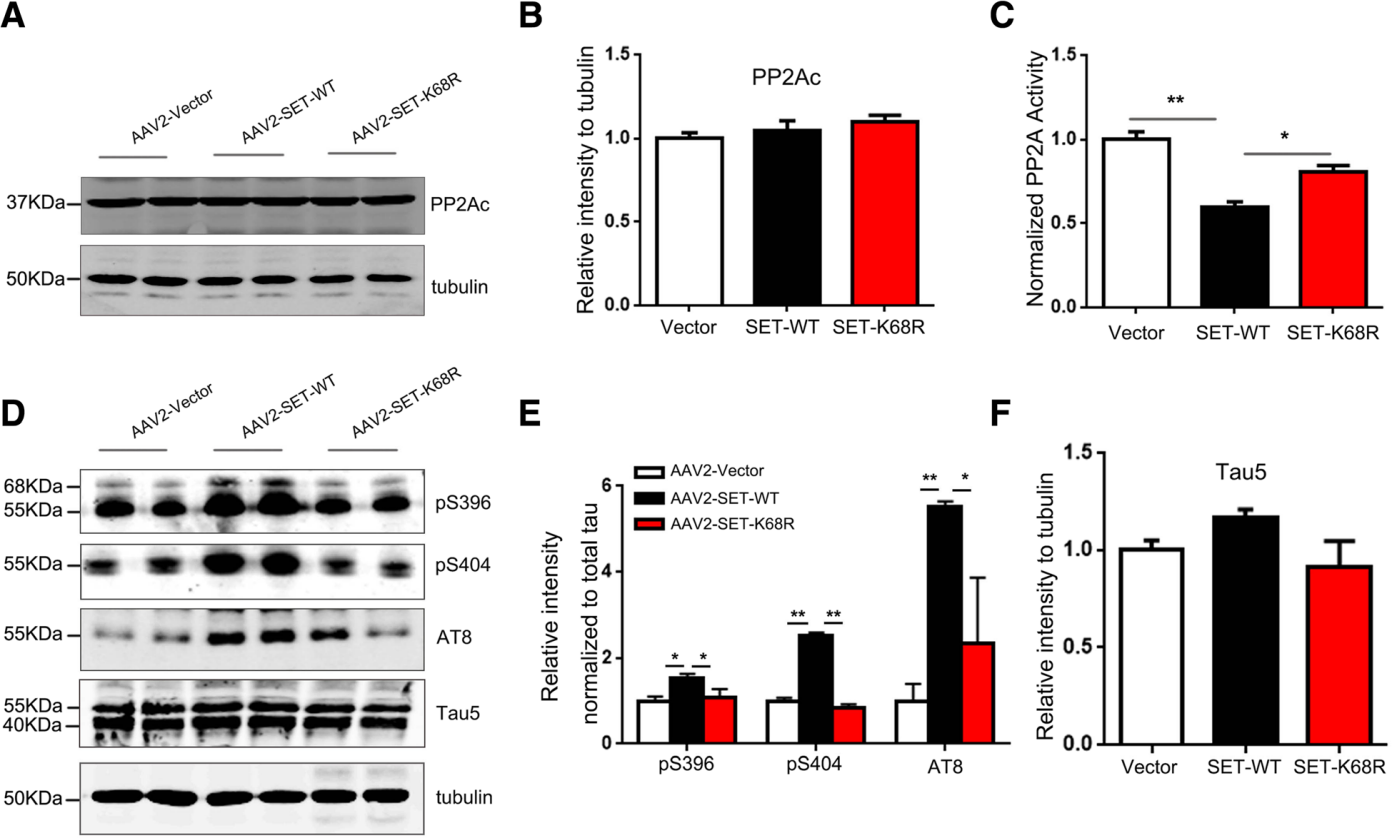
SET SUMOylation leads to PP2A inhibition and tau hyperphosphorylation. **a** Levels of PP2A in hippocampus tissue homogenates was assessed via western blotting analysis and **b** quantified using ImageJ. **c** PP2A activity in hippocampal tissue homogenates was measured using commercial PP2A assays. **d** Tau phosphorylation in hippocampal tissue homogenates was measured via western blotting analysis with the indicated panel of phosphorylation site-specific antibodies. Blots were normalized to total tau protein probed using Tau-5. Tubulin was used as a loading control. **e** Tau phosphorylation at Ser396, Ser404, AT8 (Ser202/205) was quantified using ImageJ. **f** Total tau developed with Tau5 were comparable among each group. All data shown represent the mean ± SD of 3 independent experiments. *P < 0.05, **P < 0.01

Given these findings, we explored the potential mechanisms of the AAV2-SET-WT virus on learning and memory function. We investigated the effects of SUMOylation on SET distribution and PP2A activity in the mouse brain. The results of co-immunoprecipitation experiments showed that the overexpression of AAV2-SET-WT led to a high increase in SET-SUMOylation, which was lower in SET-K68R mice (Fig. 6a, b). Nuclear/cytoplasmic fractionation showed that the SET modified by SUMO-1 was primarily distributed to the cytoplasm (Fig. 6c, e), whilst AAV2-SET-K68R was primarily retained in the nucleus (Fig. 6d, f), supporting that SET WT overexpression induces its SUMOylation and cytoplasmic retention. In the mouse hippocampus, the activity of PP2A was next assessed. We found that there was no significant change in the levels of PP2Ac in the brains either in AAV2-SET-WT or AAV2-SET-K68R mice when compared with AAV2-vector control group (Fig. 7a, b). However, the activity of PP2A in mice overexpressing AAV2-SET-WT was greatly reduced compared to the control group, whilst an increase was observed in the non-SUMOylated SET K68R group compared to AAV2-SET-WT group (Fig. 7c). We also examined the phosphorylation of tau and found that, compared with the control group, its phosphorylation at Ser396, Ser404 and AT8 (Ser202/Ser205) evidently increased in AAV2-SET-WT mice (Fig. 7d, e). Tau phosphorylation levels were substantially lower in non-SUMOylated SET-K68R mice compared to SET-WT mice, while the total levels of tau (Tau5) were comparable among each group (Fig. 7d-f). Taken together, these results confirm that SET can be modified by SUMO-1 in C57/BL6 mice. SET modified by SUMO-1 is mainly distributed in the cytoplasm and causes a significant decrease in PP2A activity, which in turn leads to hyperphosphorylation of tau.

**Fig. 8:**
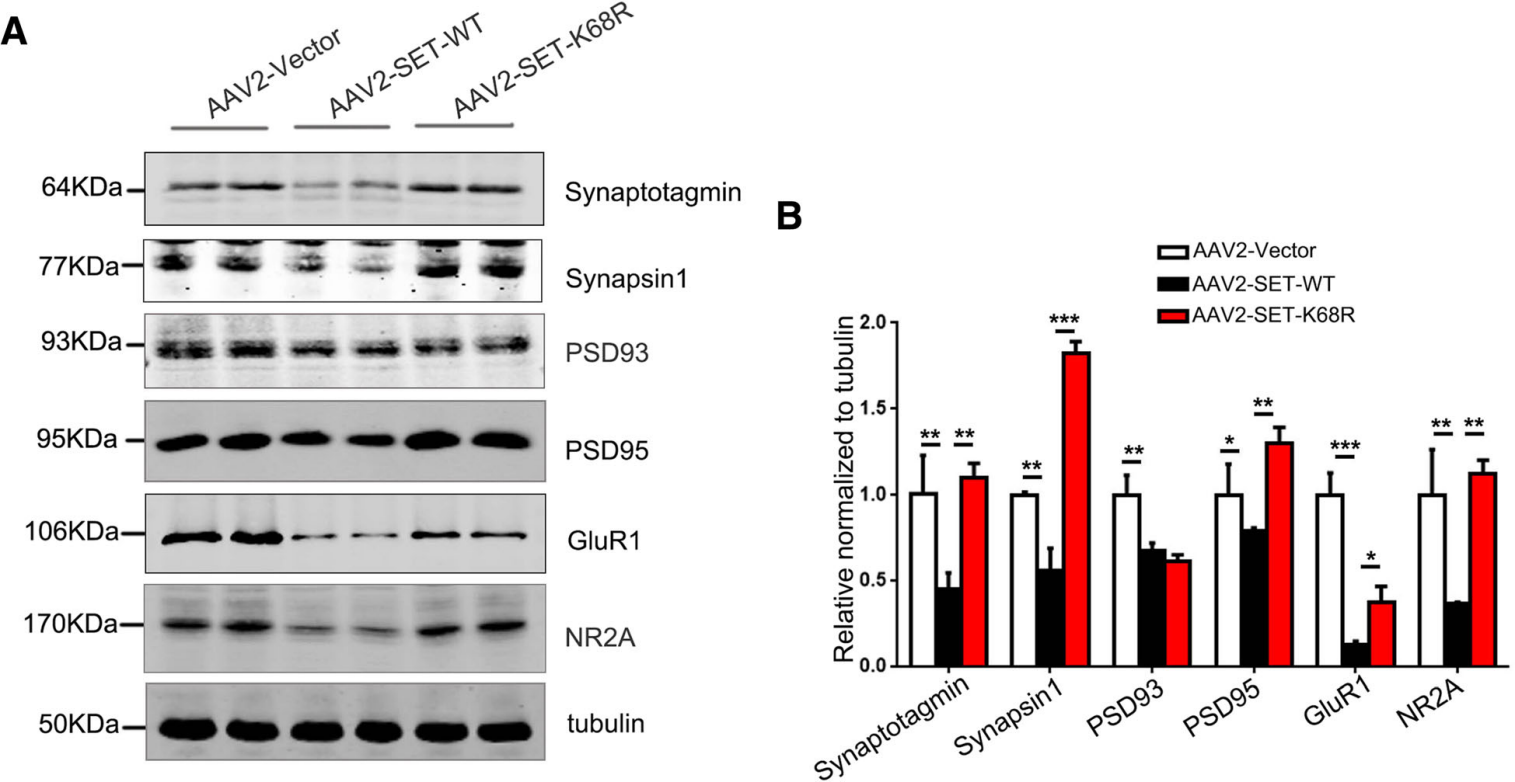
SUMOylation of SET downregulates expression of synapse related proteins. **a** Synaptotagmin, Synapsin1, PSD93, PSD95, GluR1, NR2A, were detected by western blotting analysis of hippocampus homogenates. **b** Quantification of blots in (**a**) are shown. *P < 0.05, **P < 0.01, ***P < 0.001. All the data represent the mean ± SD of 3 independent experiments

In the nervous system, synaptic function dictates the memory formation. We therefore examined synaptic protein levels in our experimental mouse models. Western blot analysis showed that AAV2-SET-WT mice displayed decreased levels of Synaptotagmin, Synapsin1, PSD93, PSD95, NR2A, GluR1 compared to the control group (Fig. 8a). However, non-SUMOylated SET-K68R recoverd the expression of these synaptic markers which were comparable to the control group (Fig. 8a). Quantification was summarized in Fig. 8b. These results demonstrate that SET-SUMOylation down-regulates the expression of synapse-associated proteins that contribute to its influence on learning and memory impairment.

### Aβ oligomers stimulation upregulates SET SUMOylation

**Fig. 9:**
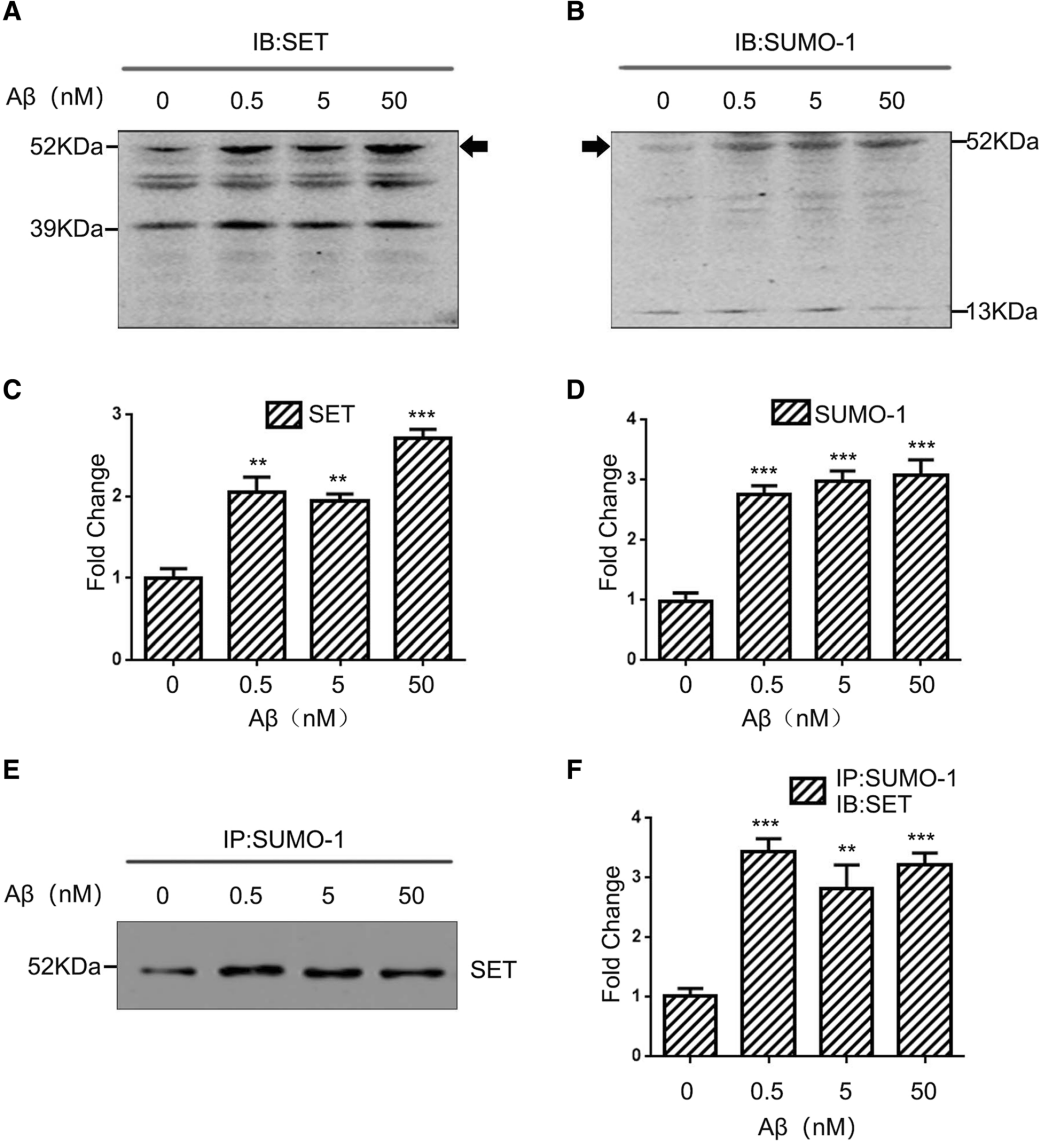
Aβ oligomers stimulation upregulates SET SUMOylation. **a** and **b** Rat primary hippocampal neurons were treated with the indicated concentrations of Aβ oligomers for 24 h. Samples were lysed with RIPA buffer and probed with anti-SET and anti-SUMO-1 antibodies via western blotting analysis. Arrows indicate 52 kDa bands that cross react with both anti-SET (39 kDa) and anti-SUMO-1 (13 kDa) representing endogenously SUMOylated SET induced by Aβ exposure. **c** and **d** Quantification of the blots in (**a** and **b**). **e** Rat primary hippocampal neurons were treated with the indicated concentrations of Aβ oligomers . Cells were lysed and immunoprecipitations performed using anti-SUMO-1 antibodies. Pull-downs were subjected to western blotting analysis and probed with anti-SET antibodies. **f** Quantification of the blots in (**e**).**P < 0.01, ***P < 0.001 vs. DMSO (0 nM Aβ). All data represent the mean ± SD of 3 independent experiments

To further explore the upstream factors of SET SUMOylation during the development of AD, we used the key pathogenic molecule Aβ to stimulate primary hippocampal neurons. Both antibodies against SET antibody (Fig. 9a) and SUMO-1 antibody (Fig. 9b) reacted with the 52 KDa bands (SET, 39 KDa; SUMO-1, 13 KDa), indicating that the 52 KDa band represented SUMOylated SET. The quantitative data were summarized in Fig. 9c (anti-SET) and Fig. 9d (anti-SUMO-1). To further confirm the 52 KDa bands, we performed co-immunoprecipitation and found that Aβ stimulation induced a marked increase of SET SUMOylation at 52 KDa bands. (Fig. 9e-f). These findings suggest that during AD, as the number of Aβ lesions increases, Aβ simultaneously promotes SET SUMOylation. This in turn would lead to the cytoplasmic retention of SET, decreased PP2A activity, in turn, abnormal hyperphosphorylation of tau, and consequently exacerbated AD progression.

## Discussion

In AD, decreased PP2A activity is considered an important factor in hyperphosphorylation of tau and the formation of NFTs [21]. SET is an endogenous inhibitor of PP2A and displays increased expression and cytoplasmic retention in the AD brain [26, 34, 36]. The mechanisms that govern SET retention in cytoplasm and how this promotes the inhibition of PP2A to cause neuronal lesions have not been defined. Our previous study showed that SET is phosphorylated at Ser9 in AD brains and phosphorylation of SET induces its cytoplasmic detention, inhibition of PP2Ac and tau hyperphosphorylation in HEK293/tau cells [39]. Moreover, we further demonstrate that CK2 phosphorylates Ser9 on SET leading to its cytoplasmic translocation and inhibition of PP2A, which subsequently results in tau phosphorylation and its neurofibrillary degeneration in vivo [40] . In the current study, we provide extensive evidence supporting that SET can be SUMOylated. Interestingly, we found K68 residue is the major SUMOylation site of SET, which is required for SET translocation from the nucleus to the cytoplasm and subsequently induces inhibition of PP2A and hyperphosphorylation of tau in HEK-293 cells. Overexpression of wild type SET but not non-SUMOylated K68R in C57/BL6 mice significantly inhibits PP2A activity, leading to tau hyperphosphorylation, less synapse loss and cognitive deficits. Together, our data strongly support the notion that SET SUMOylation promotes its cytoplasmic retention and mediates tau pathology.

As an important post-translational modification, SUMOylation is involved in almost all aspects of cell physiology and as such has been the subject of intensive research efforts. Whole-genome studies have revealed the links between the SUMO-related gene mutations and sporadic AD [13]. Genomic DNA analysis of patients with delayed-onset AD found that only intron 7 SNP (rs761059) of the unique UBC9 gene was significantly associated with the disease [4]. Given the variety of SUMOylation targets in neurons, its dysregulation in relation to AD is perhaps unsurprising. In recent years, further SUMOylation substrates have been discovered amongst which APP and tau are directly associated with AD [18, 23, 28]. With increasing SUMOylation levels, the production of Aβ increases [20, 41]. In the AD-mouse model, tau binds SUMO-1 at K340 [23, 32]. In the present study, we found that SET K68 was a potential binding site for SUMO using SUMOsp2.0 software. We identified that SET is modified by SUMO-1 at K68 in vitro and in vivo.

The transport of proteins between the nucleus and the cytoplasm occurs through nuclear pore complexes and is facilitated by numerous transport factors. These transport processes are commonly regulated by post-translational modifications. Examples of protein SUMOylation influencing nuclear-cytoplasmic transport have been documented [16], both through its effects on the physical properties of cargo molecules and by directly regulating the functions of components of the nuclear transport machinery. However, the relationship between SUMO, the nucleo-cytoplasmic transport machinery and its contribution to AD, requires further investigation. Our recent studies showed that mimicking phosphorylation at Ser9 inhibits the nuclear import of SET and thus retains the protein in the cytoplasm [39]. Furthermore, CK2 activation leads to SET Ser9 phosphorylation, resulting in its cytoplasmic translocation [40]. In the current study, we found that overexpression of wild type SET but not non-SUMOylated K68R induces SET cytoplasmic retention, which significantly inhibit PP2A activity either in HEK-293 cells or in C57/BL6 mice, resulting in tau hyperphosphorylation.

Learning and memory are highly dependent on the hippocampus [30]. Most AD patients have learning and memory impairments [1]. Meanwhile, previous study has shown that females, not males are susceptible to hormone (especially estrogens) levels, and males are more stable in behavioral study [11]. In the present study, following overexpression of AAV2-SET-WT or AAV2-SET-K68R in C57/BL6 male mice for 1 month, conditional fear testing showed no influence of SET on the percentage of freezing times at 4 h, but a significant influence on the 24-h freezing times, showing that the long-term memory capacity of the AAV-SET-WT groups was significantly lower than that of the control group. Interestingly, non-SUMOylated SET K68R mice displayed significantly higher values at the 24-h time point than the SET wild-type group. In the Morris water maze test, AAV2-SET-WT group displayed significantly lower learning and memory activity than the control group, but AAV2-SET-K68R group were significantly higher than the SET wild-type group. Previous study has found that synaptic-associated proteins are closely related to long-term memory [6], which was consistent with our findings that SET SUMOylation down-regulates the expression of synapse-associated proteins and subsequently causes learning and memory impairment.

To address the mechanism of enhanced SET SUMOylation during the AD process, we treated primary rat hippocampal neurons with Aβ, and assessed the levels of SET SUMOylation. We observed an increased SUMOylation of SET with increasing Aβ concentrations, explaining its contribution to AD progression. Together with our previous studies that Aβ also induces BACE1 SUMOylation and Tau SUMOylation [23, 28], we here speculate that Aβ mediating SET SUMOylation accelerates and aggravates the clinical course of AD.

## Conclusions

Taking these data together, we have found in the present study that SET SUMOylation promotes its cytoplasmic retention, which results in inhibition of PP2A activity and tau hyperphosphorylation. Aberrant tau then further triggers AD progression. We thus reveal the mechanistic contribution of SET SUMOylation to the AD process.

## Abbreviations

AD: Alzheimer’s disease
APP: Amyloid precursor protein
Aβ: β-Amyloid
DMEM: Dulbecco’s modified eagle’s medium
FBS: Fetal bovine serum
HEK293T: Human embryonic kidney 293
NFTs: Neurofibrillary tangles
PP2A: Protein phosphatase 2A
